# Effect of Cadmium Accumulation on the Performance of Plants and of Herbivores That Cope Differently With Organic Defenses

**DOI:** 10.3389/fpls.2018.01723

**Published:** 2018-11-28

**Authors:** Diogo Prino Godinho, Helena Cristina Serrano, Anabela Bernardes Da Silva, Cristina Branquinho, Sara Magalhães

**Affiliations:** ^1^Centro de Ecologia, Evolução e Alterações Ambientais, Faculdade de Ciências, Universidade de Lisboa, Lisbon, Portugal; ^2^Instituto de Biossistemas e Ciências Integrativas (BioISI), Lisbon, Portugal

**Keywords:** metal accumulating plants, plant defence, tomato, spider mites, elemental defence hypothesis

## Abstract

Some plants are able to accumulate in their shoots metals at levels that are toxic to most other organisms. This ability may serve as a defence against herbivores. Therefore, both metal-based and organic defences may affect herbivores. However, how metal accumulation affects the interaction between herbivores and organic plant defences remains overlooked. To fill this gap, we studied the interactions between tomato (*Solanum lycopersicum*), a model plant that accumulates cadmium, and two spider-mite species, *Tetranychus urticae* and *Tetranychus evansi* that, respectively, induce and suppress organic plant defences, measurable via the activity of trypsin inhibitors. We exposed plants to different concentrations of cadmium and measured its effects on mites and plants. In the plant, despite clear evidence for cadmium accumulation, we did not detect any cadmium effects on traits that reflect the general response of the plant, such as biomass, water content, and carbon/nitrogen ratio. Still, we found effects of cadmium upon the quantity of soluble sugars and on leaf reflectance, where it may indicate structural modifications in the cells. These changes in plant traits affected the performance of spider mites feeding on those plants. Indeed, the oviposition of both spider mite species was higher on plants exposed to low concentrations of cadmium than on control plants, but decreased at concentrations above 0.5 mM. Therefore, herbivores with contrasting responses to organic defences showed a similar hormetic response to metal accumulation by the plants. Additionally, we show that the induction and suppression of plant defences by these spider-mite species was not affected by the amount of cadmium supplied to the plants. Furthermore, the effect of cadmium on the performance of spider mites was not altered by infestation with *T. urticae* or *T. evansi*. Together, our results suggest no interaction between cadmium-based and organic plant defences, in our system. This may be useful for plants living in heterogeneous environments, as they may use one or the other defence mechanism, depending on their relative performance in each environment.

## Introduction

Plants are exposed to an array of abiotic and biotic stresses. The mechanisms that allow them to survive these adversities imply physiological and structural transformations that can be costly to the plants, affecting negatively their growth and fitness (Boyer, 1982; Wang et al., 2003). One such stress is high bioavailable metal concentrations in soil, either naturally (geochemical anomalies) or due to anthropogenic activities. Although these high concentrations are toxic to most organisms, some plant species or populations, termed metallophytes, thrive in such environments. They achieve this either by limiting the metal uptake or the translocation to the shoot (excluders), or by storing metals in their shots (accumulators; Baker, 1987). However, these strategies entail costs that may be reflected in the plant performance, namely in plant biomass, in the water content of the shoots, and/or in the root to shoot ratio (Kastori et al., 1992; Das et al., 1997; Larbi et al., 2002; Chaffei et al., 2004; Devi et al., 2007). In addition, the stress caused by metal toxicity may lead to disturbances in the carbon and nitrogen metabolism, affecting the nutritional status of various plant parts (Larbi et al., 2002; Chaffei et al., 2004; Wahid et al., 2007), and potentially changing the accumulation of soluble sugars, either leading to increased (Devi et al., 2007; Rosa et al., 2009; Mishra et al., 2014) or decreased (Scheirs et al., 2006; Shackira and Puthur, 2017) sugar concentrations in the shoots. These physiological changes in the plant may also affect the performance of the herbivores feeding on those plants (White, 1984; Scheirs et al., 2006).

Besides being costly for the plant, accumulation of some metals is highly toxic to herbivores as well. Indeed, due to their elemental nature, they cannot be degraded by chemical counter-defenses of the herbivores (Boyd, 2004). Therefore, metal accumulation by the plants may be detrimental to herbivores (Martens and Boyd, 1994; Boyd and Moar, 1999; Behmer et al., 2005; Kazemi-Dinan et al., 2014) and this accumulation has thus been suggested to serve as a defense against herbivory (Boyd, 2004; Poschenrieder et al., 2006; Hörger et al., 2013). If metal accumulation does not compromise the production of organic defenses, the combination of both defense strategies may give accumulating plants an advantage over non-accumulating competitors (Boyd, 2007). It has been shown that metal exposure may directly increase the activity of some organic plant defenses, such as proteases (Pena et al., 2006; Lin et al., 2010), having possible indirect effects on herbivores. However, because both types of defenses may be costly to the plant, the production of effective metal-based defenses may lead to fewer organic plant defenses being produced. Indeed, some studies show that metal-accumulating plants produce fewer organic defenses upon pathogen attack when they are supplied with metals (Farinati et al., 2011; Fones et al., 2013). This suggests a trade-off between metal-based and organic defenses, although more evidence is needed to establish causality and determine its prevalence.

Most herbivores induce the production of organic plant defenses (Karban and Myers, 1989; Walling, 2000; Awmack and Leather, 2002). However some are able to suppress them (Musser et al., 2002; Abramovitch et al., 2006; Sarmento et al., 2011). Likewise, metal defenses vary in their effects upon herbivores. For example, the effectiveness of metal accumulation as an anti-herbivore defense varies with herbivore feeding guilds (Jhee et al., 2005; Vesk and Reichman, 2009; Konopka et al., 2013), as well as between specialist and generalist herbivores (Kazemi-Dinan et al., 2014). However, it is yet unknown whether metal-based defenses affect differently herbivores that induce or suppress organic defenses, and this may shed new light into the study of potential interactions between metal-based and organic defenses.

The model system composed of tomato plants (*Solanum lycopersicum*, L.) and herbivorous mites is ideal to test the abovementioned issues. When growing on soils with cadmium (Cd), tomato plants show higher tolerance than other species (Bingham et al., 1974; Khan and Khan, 1983; Kuboi et al., 1986) and inclusively are able to accumulate this metal in their shoots, sometimes over the Cd-hyperaccumulation threshold (100 mg.kg^-1^; Gratão et al., 2008; López-Millán et al., 2009). Among spider mites, *Tetranychus urticae* is negatively affected by the accumulation of different metals by some host plants (Jhee et al., 2005; Quinn et al., 2010), but information concerning the effects of metals on other spider-mite species is as yet lacking. Additionally, different species within the Tetranychidae show contrasting effects on the induction of organic defenses of tomato plants. Indeed, *T. urticae* induces the production of jasmonate defenses, such as proteinase inhibitors, leading to lower performance of herbivores infesting those plants (Li et al., 2002; Ament et al., 2004; Kant et al., 2004). In contrast, *Tetranychus evansi* suppresses the production of such defenses (Sarmento et al., 2011; Alba et al., 2015), leading to higher performances of herbivores on subsequent infestations (Sarmento et al., 2011; Godinho et al., 2016). These differences allow testing the possible effect of metal accumulation on the inducibility of organic plant defenses. To this aim, we assessed the effects of Cd accumulation on the performance of tomato plants and on the spider mites that infest those plants. Additionally, we evaluated the effect of herbivory on jasmonate defenses and subsequent infestations by spider mites, on plants exposed to different Cd concentrations.

## Materials and Methods

### Biological Materials and Rearing Conditions

#### Plants

Tomato plants (*Solanum lycopersicum*, var. Moneymaker) were sowed in a climate chamber (25°C, photoperiod 16/8 h light/darkness), in a soil (pH 5.0–6.0; Siro, professional substrates, Portugal)/vermiculite mixture (4:1) and watered 3 times per week for the first 2 weeks. In the third and fourth weeks, plants were watered once a week with tap water and twice a week with 60 mL of a Cd chloride solution with two ranges of concentrations: a wide range: 0, 0.01, 0.1, 0.5, 1, 2, or 10 mM; and a narrow range: 0, 0.1, 0.25, 0.5, 0.75, 1, or 1.5 mM. Using the wide range, we tested the effects of high Cd concentrations in the plant and on spider mites. Using the narrow range allowed us to measure plant and spider-mite traits with higher resolution. At the end of the fourth week, plants were used in the experiments.

#### Spider Mites

*Tetranychus urticae* was collected from tomato plants in Portugal in 2010, and reared on bean plants (*Phaseolus vulgaris*, L.) since then (Clemente et al., 2016). In January 2016, a sub-set of the population (>300 mated females) was transferred to tomato plants and maintained on this host for six generations, before being used in the subsequent experiments. *T. evansi* was collected from *Datura stramonium*, L. in 2013, in Portugal, and reared on tomato plants ever since (Zélé et al., 2018). The two species were maintained, separately, in plastic boxes containing two entire tomato plants, in a climate chamber with conditions identical to those of the plant growing compartment (25°C, photoperiod 16/8 h light/darkness). Once a week, one plant was removed, and its leaves were cut and placed on top of the leaves of a new plant, allowing spider mites to migrate to new intact plants. To ensure that females used in the experiments were approximately of the same age, adult females where isolated on separate leaves and allowed to lay eggs for 48 h. Twelve days later, the adult females resulting from these cohorts were used in the experiments.

### General Methodology

The performance of plants and spider mites was assessed using plants exposed to both ranges of Cd supply. For every assay the plants were between 4 and 5 weeks old, but to control for the effect of leaf age, we also always used the third leaf from below (third older) for the several measurements.

#### Plant Performance

Because the plant material collected was not enough to use in every assay, measurements were performed with different plants: Plants exposed to the wide range of concentrations (0–10 mM, *N* = 6 per Cd concentration) were used to determine Cd accumulation on the leaf, as well as the amount of calcium (Ca) and magnesium (Mg). As Cd^2+^ uses the same transporters as these ions, their assimilation by the plant may be hampered by Cd, which is not the case in hyperaccumulating plants (Gomes et al., 2013). From the narrow range (0–1.5 mM), half the plants (*N* = 6 per Cd concentration) were used to obtain the biomass parameters (root/shoot; specific leaf area and water content), however, due to technical problems, the plants supplied with 1.0 mM of Cd could not be used in this assay. The remaining plants (*N* = 6 per Cd concentration) were used to measure the amount of soluble sugars and to determine the carbon (C) to nitrogen (N) ratio. Nevertheless, for each plant, and before any destructive assay, we determined the spectral reflectance of the leaf, a non-invasive method that provides a general assessment of plant stress (Carter, 1993; Carter and Knapp, 2001).

##### Spectral analysis

The spectral reflectance was measured on one leaf from each plant, five measurements per leaf, using a UniSpec spectroradiometer (PP Systems, Haverhill, MA, United States). The spectral data generated by these measurements was analyzed by calculating spectral reflectance factors (R) for each wavelength (between 300.4 and 1148.1 nm with intervals of 3.4 nm). These factors were obtained by normalizing the reflected radiation from the leaves by a reflectance white standard. Several vegetative indices can be determined using reflectance data and used as a proxy of plant stress, being the most commonly used the Normalized Difference Vegetation Index (NDVI) as it reflects the efficiency of the photosynthetic system (Sridhar et al., 2007). Therefore, we here measured NDVI ((R810–R680)/(R810+R680)). In addition, we measured the SC index, which is representative of structural changes (SC) in leaf cells caused by accumulation of Cd (R1110/R810; Sridhar et al., 2007). Moreover, as it has been proposed that plants respond similarly to UV-B light exposure and herbivory, such as producing phenolic compounds (Roberts and Paul, 2006; Izaguirre et al., 2007), we also analyzed the spectral data under those wavelengths. For that we averaged, for each plant, the spectral reflectance factors of all UV-B wavelengths (R300.4–R313.9), referred afterward simply as UV-B reflectance.

##### Cadmium, calcium, and magnesium quantification

One leaf from each plant was dried for 72 h at 60°C until constant mass and uniformly ground. The elements were then quantified using Inductively Coupled Plasma – Atomic Emission Spectrometry (ICP – AES, Agilent 7500ce – Eurofins, Spain), after nitric acid digestion, with a detection limit of 0.1 μg/L.

##### Root to shoot ratio, specific leaf area and plant water content

All leaves and roots of each plant were collected, then the area of each leaf was measured with a laboratory leaf meter (LI-COR Biosciences). Next, the fresh weight of leaves and roots was obtained. Each leaf and the roots were then separately dried for 72 h at 60°C until constant mass and again weighed. The ratio between the dry weight of the roots and the dry weight of the leaves (root/shoot) was determined as well as the specific leaf area (SLA, total leaf area/total leaf dry weight) and plant water content (fresh weight–dry weight/fresh weight).

##### Carbon to nitrogen ratio

One leaf from each plant was dried at 60°C until constant mass and again weighed. The total carbon (C) and nitrogen (N) contents (grams of C or N per 100g of leaf dry weight) of each leaf was determined by dry combustion using an elemental analyser (EuroVector, Italy; Rodrigues et al., 2009).

##### Soluble sugar contents

One leaf disk (θ 12 mm) was stored at -80°C and subsequently used to quantify the amount of soluble sugars. These were extracted from the leaf disk using 2 mL of 80% ethanol at 80°C and then quantified through changes in absorbance, at 405 nm for sucrose, using the resorcinol (1,3-dihidroxybenzene) method (de Carvalho et al., 2015), and 490 nm for glucose and fructose, using DNS (de-nitrosalicilic acid) as an oxidizing agent (Santos et al., 2017).

#### Spider-Mite Performance

Six leaf disk arenas (θ 12 mm) were cut from one leaf (third from below) of each plant (*N* = 6 per Cd concentration for the wide range, *N* = 12 per Cd concentration for the narrow range) and placed on a petri dish on top of wet cotton wool. One female spider mite of one of the two species was placed on each arena (three arenas per species per plant) and allowed to feed and oviposit for 4 days. Daily survival and fecundity of each female were recorded. The daily fecundity of spider mites was obtained by dividing the number of eggs laid by the number of days the female lived. In a previous study, it has been shown that this measurement is highly correlated with total lifetime fecundity (Clemente et al., 2018). Therefore, this measure can also be considered as an indication of the overall performance of spider mites.

#### Interaction Between Cd Accumulation and Inducibility of Jasmonate Organic Defenses

To test whether the effect of Cd and jasmonate organic defenses on herbivores are independent, tomato plants were exposed to three different Cd concentrations (0, 0.5, and 1.5 mM) as described before. Next, plants from the three treatments were infested for 48 h with either 100 *T. evansi* or *T. urticae* females on the third leaf (from below), or they were left un-infested (*N* = 12 plants per treatment; 9 treatments: 3 Cd concentrations vs. 3 infestation status – un-infested plants, plants infested with *T. urticae* and plants infested with *T. evansi*). Afterward, the plants were cleaned by removing all the mites, web, and eggs with a brush.

The performance of spider mites was determined as above.

##### Activity of trypsin inhibitors (TIs)

Plant material from the leaf used to determine the performance of spider mites was stored at -80°C and used, later, to quantify the activity of TIs, as a proxy for inducibility of the jasmonic acid pathway by spider mites (Sarmento et al., 2011; Godinho et al., 2016; Paulo et al., 2018). Approximately 300 mg of the leaf material stored at -80°C was weighed, ground, and homogenized with 600 μL of extraction buffer (0.1 M Tris-HCl, pH 8.2; 20 mM CaCl_2_; 1:3). Each sample was centrifuged at 4°C, 16.0 ×*g* for 25 min, and the supernatant was separated from the pellet and used in the spectrophotometer assay. This assay, adapted from Paulo et al. (2018) consisted in measuring the changes in absorbance at 405 nm caused by the activity of trypsin upon its substrate N-α-Benzoyl-DL-arginine 4-nitroanilide hydrochloride (BApNA).

### Statistical Analyses

All statistical analyses were performed with the software package R 3.0.2. The normality of the residuals of each model was tested using a Shapiro–Wilk normality test and, when needed, a Box-Cox transformation to the data was performed. Models were simplified by sequentially removing non-significant interactions and factors. Due to logistic constraints, each experiment was repeated in blocks of three plants per treatment. Block was thus included in the models as a random factor.

The effects of Cd exposure on NDVI, SC index (R1110/R810) and reflectance under the UV-B spectrum were determined using general linear mixed models (lmm) with, respectively NDVI, SC index or UV-B reflectance as response variables, Cd supplied as a fixed factor and block as a random factor.

The relation between the Cd contained in the solution administrated to the soil and the Cd contained in the leaves was determined with a Spearman correlation, due to the non-normality of the data. Furthermore, the relation between Cd contents and the amount of calcium (Ca) and magnesium (Mg) present on the leaves was assessed with a Pearson correlation.

The effects of Cd on specific leaf area, water and soluble sugar contents were tested using general linear mixed models (lmm) with Cd supplied as a fixed factor and block as a random factor, whereas differences in root/shoot and in C/N were determined using a generalized linear mixed model (glmm) with a binomial distribution, and the same factors as above.

The effects of Cd on daily fecundity of spider mites were determined for each range, using general linear mixed models (lmm) with species tested and Cd supplied as fixed factors and block as a random factor. Additionally, because the soluble sugar contents and the spectral SC index (R1110/R810) were affected by Cd, we tested whether changes in those traits influenced the daily fecundity of spider mites using a multivariate analysis of variance with distance matrices (adonis function, vegan package; Oksanen et al., 2013). The fecundity of *T. evansi* and *T. urticae* were used as response variables, the amount of sucrose and glucose plus fructose or the spectral SC index (R1110/R810), were used as fixed factors.

The statistical analysis of the interactions between Cd accumulation and jasmonate organic defenses were performed using general linear mixed models (lmm) with daily fecundity of *T. evansi* or the amount of trypsin inhibited as response variables, Cd supplied (0 mM; 0.5 mM; 1.5 mM) and infestation status (un-infested plants; plants previously infested with *T. urticae*; plants previously infested with *T. evansi*) used as fixed factors and block as a random factor.

## Results

### Effect of Cd on the Performance of Tomato Plants

Cadmium exposure had no effect on NDVI (Table 1). However, significant differences were detected for the SC index (R1100/R810), (Table 1) on plants exposed to 2 mM or 10 mM of Cd (Table 2), suggesting structural changes in the leaf cells. The same pattern was detected when analyzing the narrow range of Cd concentrations (Table 1) but only for plants exposed to 1 mM and not for plants exposed to 1.5 mM (Table 2). Additionally, the UV-B reflectance of plants was significantly affected by Cd exposure (Table 1), for concentrations higher than 1 mM in the wide range (Table 2) and higher than 0.75 mM for the narrow range (Table 2).

**Table 1:** Statistical analyses of the effect of cadmium on leaf reflectance.

Variable of interest	Data subset	Exploratory variable	*Df*	*F*	*P*-value
NDVI	Wide range	Cd supplied	6	0.21	0.97
	Narrow range		5	0.83	0.53
SC index	Wide range	Cd supplied	6	3.19	**0.015**
	Narrow range		5	4.49	**0.001**
UV-B reflectance	Wide range	Cd supplied	6	11.44	**<0.001**
	Narrow range		5	10.15	**<0.001**

*p-values < 0.05 are indicated in bold.*

**Table 2:** *A posteriori* contrasts on the effect of cadmium on leaf reflectance.

Variable of interest	Data subset	Contrast	*Z*-value	*P*-value
SC index	Wide range	0 mM vs. 0.01 mM	1.23	0.88
		0 mM vs. 0.1 mM	1.80	0.54
		0 mM vs. 0.5 mM	2.87	0.06
		0 mM vs. 1 mM	-1.89	0.49
		0 mM vs. 2 mM	-0.18	**0.001**
		0 mM vs. 10 mM	-0.13	**0.008**
	Narrow range	0 mM vs. 0.1 mM	-0.79	0.97
		0 mM vs. 0.25 mM	-0.20	0.99
		0 mM vs. 0.5 mM	0.84	0.96
		0 mM vs. 0.75 mM	-3.38	**0.009**
		0 mM vs. 1.5 mM	-1.43	0.71
UV-B reflectance	Wide range	0 mM vs. 0.01 mM	1.67	0.64
		0 mM vs. 0.1 mM	2.12	0.34
		0 mM vs. 0.5 mM	3.91	**0.002**
		0 mM vs. 1 mM	-0.11	**<0.001**
		0 mM vs. 2 mM	-0.13	**<0.001**
		0 mM vs. 10 mM	-0.17	**<0.001**
	Narrow range	0 mM vs. 0.1 mM	1.50	0.66
		0 mM vs. 0.25 mM	2.35	0.17
		0 mM vs. 0.5 mM	4.07	**<0.001**
		0 mM vs. 0.75 mM	-5.28	**<0.001**
		0 mM vs. 1.5 mM	-5.74	**<0.001**

*p-values < 0.05 are indicated in bold.*

**FIGURE 1:**
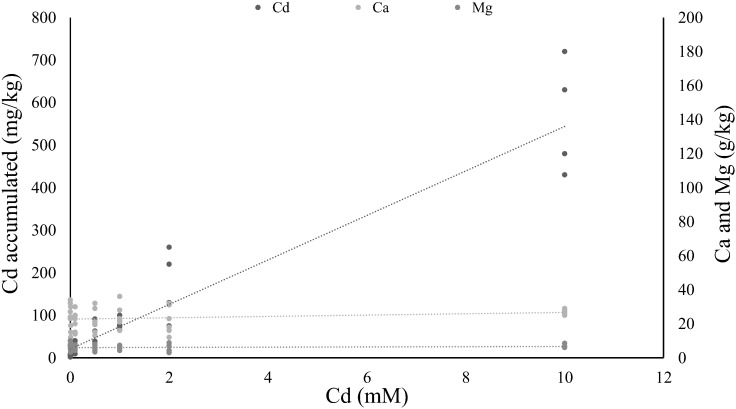
Relation between cadmium supplied in soil solution in relation to cadmium, calcium, and magnesium concentration on tomato leaves. Lines represent linear regressions between the concentration of cadmium, calcium or magnesium solutions supplied to the plant (0, 0.01, 0.1, 0.5, 1, 2, and 10 mM; six plants per concentration) and the cadmium accumulated on the leaves, or the calcium and magnesium present on those leaves.

The concentration of Cd accumulated in tomato leaves correlated positively with the Cd concentrations that plants were exposed to, in a linear way (*y* = 52.299*x* + 21.165, rho = 0.945, *P* < 0.001; Figure 1). The amount of Ca and Mg in the leaves did not change significantly with Cd accumulation (*R*^2^= 0.25, *P* = 0.11 for Ca and *R*^2^ = 0.23, *P* = 0.14 for Mg).

**FIGURE 2:**
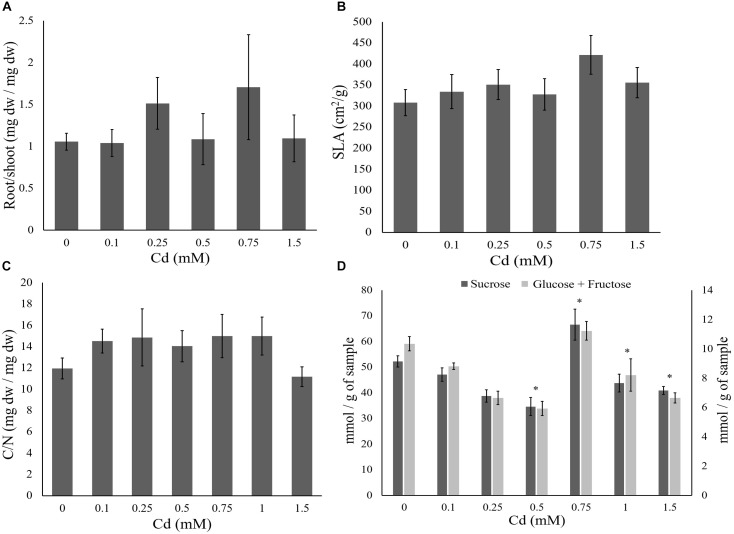
Effect of cadmium on the performance of tomato plants. Tomato plants were supplied with different cadmium concentrations (0, 0.1, 0.25, 0.5, 0.75, 1, or 1.5 mM). The plant traits (±standard error – vertical bars; six plants per concentration) were: **(A)** average root to shoot ratio; **(B)** average specific leaf area (SLA, cm^2^/g); **(C)** average carbon to nitrogen ratio of the leaves; **(D)** average glucose and fructose (light gray bars) and sucrose (dark gray bars) concentration (mmol per gram of leaf fresh weight). ^∗^Represents significant differences from the control plants.

Cadmium supplied to plants did not significantly affect water content of the leaves, SLA and root/shoot ratio (Tables 3, 4 and Figures 2A,B). No effect of Cd exposure was observed in the C/N content of the tomato leaves up to 1.5 mM (Table 3 and Figure 2C). However, the amount of soluble sugars in the leaves was affected by the concentration of Cd to which plants were exposed (Table 3 and Figure 2D). The amount of both sucrose and glucose plus fructose decreased in plants exposed to low concentrations of Cd, having the lower values at 0.5 mM (Table 5 and Figure 2D). In plants exposed to 0.75 mM of Cd, the levels of sugars peaked to values higher than in control un-exposed plants but then decreased again for higher concentrations to values lower than on control plants (Table 5 and Figure 2D).

**Table 3:** Statistical analyses of the effects of cadmium on plant performance traits.

Variable of interest	Explanatory variable	*df*	χ^2^/*F*	*P*-value
Water content	Cd supplied	5	34.03	0.31
SLA (specific leaf area)	Cd supplied	5	1.70	0.16
Root/shoot	Cd supplied	5	34.01	0.81
C/N	Cd supplied	6	4.42	0.65
Sucrose	Cd supplied	6	9.77	**<0.001**
Glucose + fructose	Cd supplied	6	13.21	**<0.001**

*p-values < 0.05 are indicated in bold.*

**Table 4:** Effect of cadmium on plant biomass.

Biomass	Contrast	Fresh weight (mg ± SE)	Dry weight (mg ± SE)
Shoots	0 mM	48.44 ± 0.35	5.34 ± 0.06
	0.1 mM	52.23 ± 0.61	5.23 ± 0.09
	0.25 mM	41.52 ± 0.68	4.48 ± 0.09
	0.5 mM	47.00 ± 0.48	5.23 ± 0.08
	0.75 mM	37.04 ± 0.76	3.65 ± 0.09
	1.5 mM	44.83 ± 0.54	4.41 ± 0.08
Roots	0 mM	91.34 ± 0.94	5.56 ± 0.07
	0.1 mM	79.23 ± 0.90	5.00 ± 0.08
	0.25 mM	88.87 ± 0.89	5.79 ± 0.08
	0.5 mM	100.41 ± 1.04	5.00 ± 0.09
	0.75 mM	78.48 ± 0.62	4.73 ± 0.13
	1.5 mM	72.98 ± 0.80	4.36 ± 0.08

*Average biomass of plants exposed to the narrow range of cadmium (N = 6).*

**Table 5:** *A posteriori* contrasts for the effects of cadmium on soluble sugar contents.

Variable of interest	Contrast	*t*-Value	*P*-value
Sucrose	0 mM vs. 0.1 mM	-1.07	0.29
	0 mM vs. 0.25 mM	-2.81	**0.008**
	0 mM vs. 0.5 mM	-3.67	**<0.001**
	0 mM vs. 0.75 mM	2.98	**0.005**
	0 mM vs. 1.5 mM	-2.36	**0.024**
Glucose + fructose	0 mM vs. 0.1 mM	1.61	0.12
	0 mM vs. 0.25 mM	4.72	**<0.001**
	0 mM vs. 0.5 mM	-6.06	**<0.001**
	0 mM vs. 0.75 mM	-0.79	**0.043**
	0 mM vs. 1.5 mM	-4.59	**<0.001**

*p-values < 0.05 are indicated in bold.*

### Effect of Cd Accumulation on the Performance of Spider Mites

**FIGURE 3:**
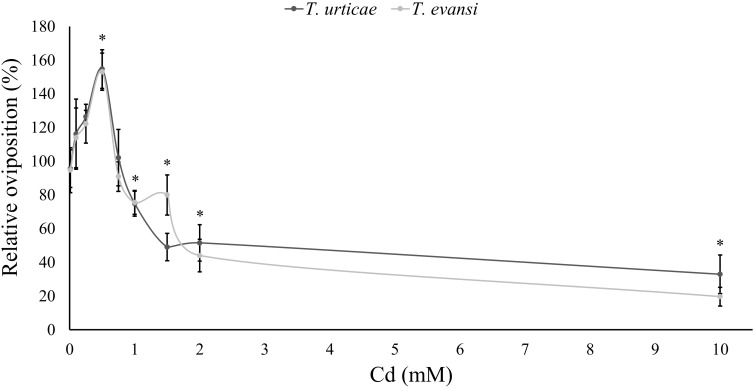
Performance of spider mites on leaves of tomato plants exposed to cadmium. Average relative oviposition rate of *T. evansi* (light gray) and *T. urticae* (dark gray) females on tomato plants (±standard error – vertical bars; 6/12 plants, 3 disks per species per plant). For each range of cadmium solutions, a (0, 0.01, 0.1, 0.5, 1, 2, or 10 mM, *N* = 6) and b (0, 0.1, 0.25, 0.5, 0.75, 1, or 1.5 mM, *N* = 12) the oviposition of spider mites was normalized to the control (no cadmium) and merged in the same panel. ^∗^Represent significant differences to the control.

The oviposition of spider mites on leaf disks was significantly affected by the Cd supplied to the plants used to make those disks (Table 6 and Figure 3). Additionally, both spider-mite species were similarly affected by the Cd concentration that plants were exposed to (Table 6). Both species increased their oviposition with low amounts of Cd until a threshold concentration, 0.5 mM (Table 6 and Figure 3). From this concentration onward, Cd had a negative effect on the oviposition rate of spider mites, reaching, values lower than in control plants at 2 mM for the wide range and at 1.5 mM for the narrow range, respectively (Table 6 and Figure 3).

**Table 6:** Statistical analyses on the effect of cadmium on the performance of spider mites.

Variable of interest	Data subset	Explanatory variable	*df*	*F*	*P*-value
Daily fecundity	Wide range	Cd supplied × tested species	6	47.04	0.14
		Cd supplied	6	64.13	**<0.001**
Daily fecundity	Narrow range	Cd supplied × tested species	6	0.12	0.99
		Cd supplied	6	10.62	**<0.001**
**Variable of interest**	**Data subset**	**Contrast**		***Z*-value**	***P*-value**
Daily fecundity	Wide range	0 mM vs. 0.01 mM	–	0.32	0.99
		0 mM vs. 0.1 mM		-0.94	0.96
		0 mM vs. 0.5 mM	–	-3.14	**0.028**
		0 mM vs. 1 mM		-1.34	0.83
		0 mM vs. 2 mM	–	-3.99	**0.012**
		0 mM vs. 10 mM	–	-4.82	**<0.001**
Daily fecundity	Narrow range	0 mM vs. 0.1 mM	–	-0.58	0.99
		0 mM vs. 0.25 mM	–	-1.45	0.77
		0 mM vs. 0.5 mM	–	-3.26	**0.018**
		0 mM vs. 0.75 mM	–	0.50	0.99
		0 mM vs. 1 mM	–	-1.43	0.78
		0 mM vs. 1.5 mM	–	3.93	**0.001**

*p-values < 0.05 are indicated in bold.*

The amount of sucrose in the leaves did not affect the fecundity of spider mites (*F*_1_ = 0.008, *P* = 0.71). In contrast, the amount of glucose plus fructose affected this trait (*F*_1_ = 0.19, *P* = 0.003). Additionally, the reflectance SC index (R1110/R810) affected the fecundity of spider mites (*F*_1_ = 0.07, *P* = 0.005).

### Interaction Between Cd Accumulation and Inducibility of Jasmonate Organic Defenses

**FIGURE 4:**
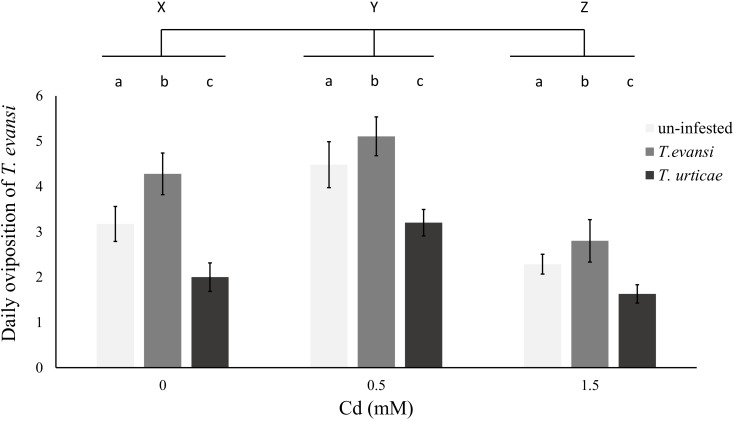
Effect of cadmium exposure and herbivory on the performance of subsequent infestations. Average number of eggs laid per day by *T. evansi* females on un-infested plants (light gray), plants infested with 100 *T. evansi* females (gray) or with 100 *T. urticae* females (dark gray) for 48 h. Plants (±standard error – vertical bars; 12 plants, 3 disks per species per plant) were exposed to a range of cadmium concentrations (0, 0.5, or 1.5 mM). Small case letters (a,b,c) represent significant differences between infestation treatments and upper case letters (X,Y,Z) between cadmium treatments, there were no significant interactions between the two factors.

The oviposition rate of *T. evansi* was affected by both Cd concentration and previous infestation with conspecifics or heterospecifics (Table 7 and Figure 4). However, the interaction between these factors was not significant (Table 7). The oviposition rate of *T. evansi* increased with previous infestation by conspecifics and decreased with previous infestation by *T. urticae* (Table 7 and Figure 4), independently of the concentration of Cd to which plants were exposed before. Moreover, the oviposition rate of *T. evansi* increased on plants exposed to 0.5 mM of Cd and decreased on plants exposed to 1.5 mM of Cd (Table 7 and Figure 4), compared to control plants, as observed in the previous results (Figure 3).

**Table 7:** Statistical analyses of the effect of cadmium and spider mite infestation on daily fecundity and concentration of trypsin inhibitors.

Variable of interest	Explanatory variable	*df*	*F*	*P*-value
Daily fecundity	Cd supplied x infestation status	4	0.47	0.76
	Infestation status	2	27.56	**<0.001**
	Cd supplied	2	32.18	**<0.001**
Trypsin inhibitors	Cd supplied × infestation status	4	0.03	0.97
	Infestation status	2	4.49	**0.014**
	Cd supplied	2	0.77	0.38
**Variable of interest**	**Contrast**		***Z*-value**	***P*-value**
Daily fecundity	Un-infested vs. *T. evansi*	–	-3.22	**0.021**
	Un-infested vs. *T. urticae*	–	3.49	**0.009**
	0 mM vs. 0.5 mM	–	4.19	**<0.001**
	0 mM vs. 1.5 mM	–	-3.88	**<0.001**
Trypsin inhibitors	Un-infested vs. *T. evansi*	–	-3.26	**0.018**
	Un-infested vs. *T. urticae*	–	3.93	**0.021**

*p-values < 0.05 are indicated in bold.*

**FIGURE 5:**
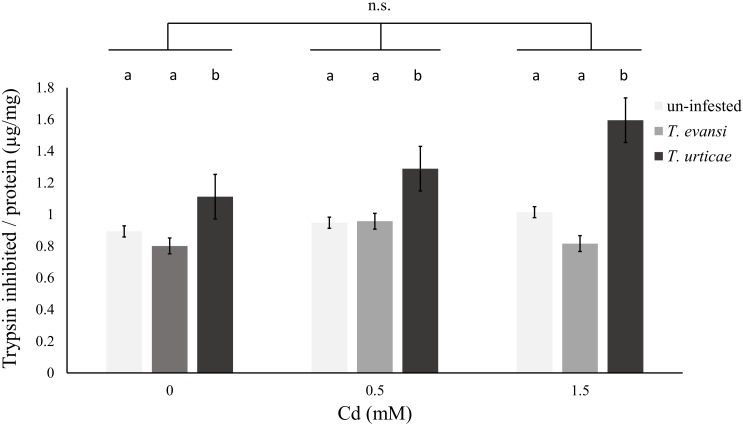
Effect of cadmium exposure and herbivory on organic plant defenses. Amount (μg) of trypsin inhibited per mg of protein in leaf samples of un-infested plants (light gray), plants infested by 100 *T. evansi* females (gray) for 48 h or 100 *T. urticae* females (dark gray) for 48 h. Plants (±standard error – vertical bars; 12 plants, 3 disks per species per plant) were exposed to a range of Cd concentrations (0, 0.5, or 1.5 mM). Lower case letters (a,b) represent significant differences between infestation treatments, Cd supplied had no significant effect, neither was the interaction between the two factors significant.

Additionally, the activity of trypsin inhibitors was modified by infestation by spider mites, independently of the concentration of Cd supplied to the plants (Table 7 and Figure 5). Cadmium accumulation did not significantly affect the activity of trypsin inhibitors (Table 7 and Figure 5).

## Discussion

Our results show that within the tested ranges, Cd exposure did not affect tomato growth (specific leaf area, root/shoot, water content, NDVI). However, variables related to leaf structure (SC index) or sugar content were affected, suggesting structural and biochemical changes in leaf cells. Spider mites infesting those plants were affected by Cd concentrations, albeit in a non-linear way. Indeed, both spider mite species had increased performance on plants mildly exposed to Cd, as compared to un-exposed plants, but lower performance after a given threshold, revealing a hormetic effect, which is a dose response phenomenon with stimulatory effects of mild concentrations and inhibitory effects at higher concentrations (Calabrese and Blain, 2009). Finally, the interaction of both spider mites with jasmonate defenses was not affected by the level of Cd that the plants were exposed to. Together, these results suggest that metal accumulation and the production of the studied plant organic defenses against herbivores do not interact with each other.

Studies regarding Cd accumulation by tomato plants reveal high variability in this trait (Hartke et al., 2013), with some plants accumulating amounts below the hyperaccumulation threshold (<100 mg/kg, Pollard, 2000), even at high concentrations of Cd supply (Ammar et al., 2007, 2008) and others accumulating above this threshold (Dong et al., 2006; Gratão et al., 2008; López-Millán et al., 2009). Here we observe that Cd accumulated linearly in the leaves of tomato plants, up to values above the hyperaccumulation threshold, suggesting that *Moneymaker*, the variety of tomato used in our study, is as a facultative hyperaccumulator. This is further confirmed by the values of Ca and Mg in the leaves which remain stable with increasing Cd in the leaves, as seen for other hyperaccumulator plants (Gomes et al., 2013; Pereira et al., 2017).

Here we report the absence of an immediate negative impact on plant growth, although there was an effective uptake of Cd into the leaves. Moreover, we also observe no differences in the carbon to nitrogen ratio in leaves of plants exposed to different Cd concentrations, indicating no shifts in the growth/defense balance (Herms and Mattson, 1992). This contrasts with previous studies showing a negative impact of Cd on tomato plant growth, for Cd accumulation values within the ranges used here (Dong et al., 2006; Ammar et al., 2007; Gratão et al., 2008; López-Millán et al., 2009). Possibly, the variety of tomato we used in this experiment is more tolerant to Cd than most other varieties. Indeed, the few studies using this variety observe no signs of toxicity (Petit and Van de Geijn, 1978; Petit et al., 1978). Another possibility is that the growing substrate affected these results. Indeed, most studies on this topic used continuously aerated hydroponics, creating an artificial situation for the plants such as the absence of microbiota around the root, and here we used soil as a substrate as in natural conditions. Growing in soils may be advantageous to plants, given that soil microbiota may regulate the process of metal accumulation in the shoots (de Souza et al., 1999; Farinati et al., 2009), reducing the costs involved in this process for the plant (Farinati et al., 2009).

In contrast to most plant traits that did not respond to Cd, we found changes in soluble sugar contents and leaf reflectance. Changes in the amount of soluble sugars in the leaves with Cd were non-linear. Soluble sugars are generally associated with an initial response to plant stress, with changes in their accumulation, either increasing or decreasing, affecting the REDOX reactions originated by environmental stress (Couée et al., 2006). Cadmium supply may lead to either an increase (Mishra et al., 2014) or a decrease (Scheirs et al., 2006; Shackira and Puthur, 2017) in the amount of soluble sugars in the shoots of exposed plants. The fluctuations we observe in the soluble sugars content may indicate that these are being affected by different processes in the plant, and this may help to reconcile the contrasting observations in the literature. Additionally, plants may also have a hormetic response to abiotic stressors, increasing their performance with small amounts of Cd until a threshold where the negative effects caused by this metal exceed the positive ones (Siddhu et al., 2008; Poschenrieder et al., 2013). If this is the case, the response of the plant to this stress may be different below and above this threshold, and this in turn could be reflected in the soluble sugar content. Corroborating this hypothesis requires more controlled experiments and a systematic measurement of Cd concentrations in the leaves. Moreover, higher concentrations of Cd exposure caused changes in leaf reflectance (SC index), which have been linked to structural changes on their leaf cells (Sridhar et al., 2007). Additionally, plants exposed to the higher concentrations of Cd showed a significant increase in the reflectance of UV-B light, which is possibly related to the production of phenolic compounds known to protect plants against abiotic stresses (Roberts and Paul, 2006; Izaguirre et al., 2007). Our results thus highlight the need to collect different measures of plant performance in response to a single abiotic stress.

**FIGURE 6:**
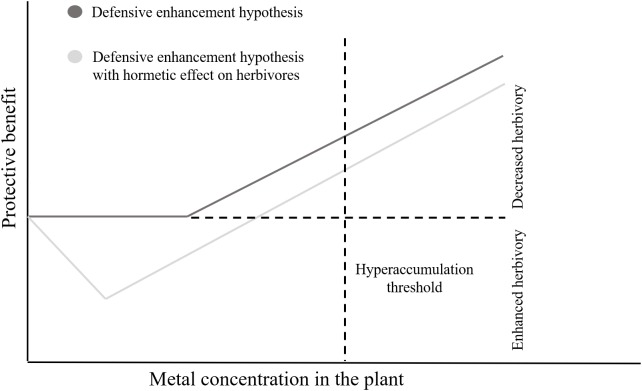
The impact of hormesis on the protective effect of metals. Schematic comparison of the protective benefit of metal accumulation to the plant between the defensive enhancement scenario and a scenario with a hormetic effect of the metal on herbivores.

The performance of spider mites was also affected by Cd accumulation in tomato leaves. Both species had a non-linear, hormetic response to this metal. Most herbivores are negatively affected by metal accumulation in the leaves of their host plants (Hanson et al., 2003; Freeman et al., 2007; Quinn et al., 2010; Stolpe et al., 2017). Still, there are some examples of higher abundances of herbivores on sites with intermediate concentrations of toxic metals (Zvereva et al., 1995; Kozlov, 2003), under natural conditions. Nevertheless, this is, to our knowledge, the first report of a hormetic effect of metals on herbivore performance. This phenomenon may significantly affect the evolution of metal accumulation, as selection will favor plants that accumulate amounts of metal above the threshold for herbivore inhibition. Such pattern may complement the “defense enhancement hypothesis” (Boyd, 2007). Indeed, given the hormetic effect of metals upon herbivores, plants are expected to reach the protective threshold of metal accumulation only when the costs of accumulation (i.e., a positive effect of metals upon herbivores) are surpassed by the benefits of metals reducing herbivory (Boyd, 2012; Figure 6). This hormetic pattern may be due to direct effects of the metal on the spider mites, or indirectly, through changes in plant quality. We observed no effect of Cd on plant biomass and C/N ratio, however, the amount of soluble sugars in the leaf significantly affected the performance of spider mites. The performance of spider mites has been reported as positively (Ximénez-Embún et al., 2016, 2017) or negatively (Wermelinger et al., 1985; Joutei et al., 2000; Scheirs et al., 2006) correlated to sugar content, indicating that this may depend on the host plant species or on other physiological responses that were not assessed. Although this correlation does not imply causation, it does suggest that Cd may indirectly affect mite performance via an effect on sugars or other physiological changes associated with them, a hypothesis that requires further tests. Additionally, we observed that the daily fecundity of both species correlated with the leaf spectral SC index, indicating a possible effect of structural changes in leaf cells caused by Cd. Still, our experiments do not exclude a possible direct effect of the metal on the performance of spider mites. Whether these correlations imply causality is a relevant question that calls for future studies.

The similarity in the hormetic pattern of the two spider mite species suggests they both may prefer to establish on plants with intermediate Cd concentrations rather than on un-contaminated plants. Moreover, their higher oviposition probably indicates that their growth rate is higher on those plants (Clemente et al., 2018). This may entail a faster saturation of that environment, relative to others. If this hormetic effect is extended to other herbivore and pathogenic species, then plants are expected to pay a high cost of mild Cd accumulation. Thus, given enough time, plants may be selected to “avoid” the level of Cd accumulation that results in better performance for the herbivores, being selected to accumulate higher amounts of metal, becoming hyperaccumulators, or to not accumulate metal at all, becoming excluders.

Because the two spider-mite species have dissimilar interactions with jasmonate organic defenses, the fact that they perform best on plants with the same Cd supply also suggests no interaction between metal accumulation and the inducibility of jasmonate defenses. Furthermore, the contrasting effects of these two spider-mite species on the activity of trypsin inhibitors and its effect on subsequent infestations were consistent across Cd environments, revealing no interference of Cd on protease activity, in contrast to what was seen in other plant species (Pena et al., 2006; Lin et al., 2010). The plants used in these experiments showed little evidence of Cd toxicity. Possibly, the effect of Cd was not strong enough to induce the protective protease activity reported for other plants (Pena et al., 2006; Lin et al., 2010). Additionally, the effect of metal supply on spider mite performance was not affected by previous infestation. Together, these results suggest that metal based and organic plant defenses do not interfere with each other, serving the same purpose. Although some studies reveal that the expression of organic defenses is lower with high metal supply (Davis et al., 2001; Tolra et al., 2001; Farinati et al., 2009, 2011; Sun et al., 2009; Fones et al., 2013; Stolpe et al., 2017; Tewes et al., 2018), how herbivores are affected by the interaction between metal accumulation and organic defenses remains poorly studied (but see Stolpe et al., 2017). Still, if metal accumulation provides the same function as organic defenses, and if the production of organic defenses is costly, this may select for a reduction in organic defenses in plants under high metal supply. The opportunity for such selection to be effective is much higher on obligate metal accumulators (Poschenrieder et al., 2006), which is not the case of tomato. Alternatively, plants may be suffering from metal accumulation, hence they may lack the necessary resources to trigger organic defenses (Farinati et al., 2009, 2011; Fones et al., 2013). As we did not observe negative effects of Cd on plant growth, it may be that cost on tomato plants due to metals was not sufficient to lead to a trade-off between these two types of defenses. Possibly, long-term exposure to this contaminant, or exposure to higher concentrations, would cause significant costs to the plant affecting its growth rate or posing constrains in fruit production. Still, a recent field work found little evidence for trade-offs between organic and inorganic defenses (Kazemi-Dinan et al., 2015).

Another possible explanation for the absence of a trade-off in our study is that the effect of metal accumulation on herbivores was non-linear. Thus, if plants would produce fewer organic plant defenses, as metal accumulation increased, herbivores would have an extra-advantage at intermediate metal concentrations, benefiting from both a high performance in response to metals, and a low exposure to organic defenses. This, in turn, would pose a strong selective pressure upon plants not to shut down organic defenses. In the absence of an interaction between metal-based and organic defenses, plants occurring in heterogeneous environments may fine tune these strategies depending on their relative performance in each environment. Possibly, plants accumulate more metal when exposed to herbivores that suppress their organic defenses, overcoming the positive effects that low concentrations may have on these herbivores. This hypothesis awaits to be tested.

In sum, our results show that spider mites with different effects on the organic defenses of tomato plants have a similar hormetic response to Cd accumulation. This suggests that the community of spider mites on tomato plants will be similar in contaminated and un-contaminated soils. Our results highlight the importance of studying the interactive effects of metal based and organic plant defenses on herbivores, using metal concentrations below the hyperaccumulating threshold, which allows using more facultative accumulator species, including some of agricultural importance, such as tomato plants.

## Data Availability

All data used in this work is archived in Dryad at doi: 10.5061/dryad.f274gs3.

## Author Contributions

DG, SM, and CB conceived the study. DG, SM, and CB designed the experiments with help from HS and ADS. DG collected the data with assistance from HS and ADS. DG analyzed the data with help from all authors. DG and SM led the writing of the manuscript, with significant help from CB and contributions by all authors. All authors gave final approval for publication.

## Conflict of Interest Statement

The authors declare that the research was conducted in the absence of any commercial or financial relationships that could be construed as a potential conflict of interest.

## References

[B1] Abramovitch R. B. Anderson J. C. Martin G. B. (2006). Bacterial elicitation and evasion of plant innate immunity. *Nat. Rev. Mol. Cell Biol.* 7 601–611. 10.1038/nrm1984 16936700 PMC2842591

[B2] Alba J. M. Schimmel B. C. Glas J. J. Ataide L. Pappas M. L. Villarroel C. A. (2015). Spider mites suppress tomato defenses downstream of jasmonate and salicylate independently of hormonal crosstalk. *New Phytol.* 205 828–840. 10.1111/nph.13075 25297722 PMC4301184

[B3] Ament K. Kant M. R. Sabelis M. W. Haring M. A. Schuurink R. C. (2004). Jasmonic acid is a key regulator of spider mite-induced volatile terpenoid and methyl salicylate emission in tomato. *Plant Physiol.* 135 2025–2037. 10.1104/pp.104.048694 15310835 PMC520773

[B4] Ammar W. B. Nouairi I. Zarrouk M. Ghorbel M. H. Jemal F. (2008). Antioxidative response to cadmium in roots and leaves of tomato plants. *Biol. Plant.* 52 727–731. 10.1007/s10535-008-0140-2

[B5] Ammar W. B. Nouairi I. Zarrouk M. Jemal F. (2007). Cadmium stress induces changes in the lipid composition and biosynthesis in tomato (*Lycopersicon esculentum* Mill.) leaves. *Plant Growth Regul.* 53 75–85. 10.1007/s10725-007-9203-1

[B6] Awmack C. S. Leather S. R. (2002). Host plant quality and fecundity in herbivorous insects. *Annu. Rev. Entomol.* 47 817–844. 10.1146/annurev.ento.47.091201.145300 11729092

[B7] Baker A. J. M. (1987). Metal tolerance. *New Phytol.* 106 93–111. 10.1111/j.1469-8137.1987.tb04685.x

[B8] Behmer S. T. Lloyd C. M. Raubenheimer D. Stewart Clark J. Knight J. Leighton R. S. (2005). Metal hyperaccumulation in plants: mechanisms of defence against insect herbivores. *Funct. Ecol.* 19 55–66. 10.1111/j.0269-8463.2005.00943.x

[B9] Bingham F. T. Page A. L. Mahler R. J. Ganje T. J. (1974). Growth and cadmium accumulation of plants grown on a soil treated with a cadmium-enriched sewage sludge. *J. Environ. Qual.* 4 207–211. 10.2134/jeq1975.00472425000400020015x

[B10] Boyd R. S. (2004). Ecology of metal hyperaccumulation. *New Phytol.* 162 563–567. 10.1111/j.1469-8137.2004.01079.x 33873764

[B11] Boyd R. S. (2007). The defense hypothesis of elemental hyperaccumulation: status, challenges and new directions. *Plant Soil* 293 153–176. 10.1007/s11104-007-9240-6

[B12] Boyd R. S. (2012). Plant defense using toxic inorganic ions: conceptual models of the defensive enhancement and joint effects hypotheses. *Plant Sci.* 195 88–95. 10.1016/j.plantsci.2012.06.012 22921002

[B13] Boyd R. S. Moar W. J. (1999). The defensive function of Ni in plants: response of the polyphagous herbivore *Spodoptera exigua* (Lepidoptera: Noctuidae) to hyperaccumulator and accumulator species of *Streptanthus* (Brassicaceae). *Oecologia* 118 218–224. 10.1007/s004420050721 28307697

[B14] Boyer J. S. (1982). Plant productivity and environment. *Science* 218 443–448. 10.1126/science.218.4571.443 17808529

[B15] Calabrese E. J. Blain R. B. (2009). Hormesis and plant biology. *Environ. Pollut.* 157 42–48. 10.1016/j.envpol.2008.07.028 18790554

[B16] Carter G. A. (1993). Responses of leaf spectral reflectance to plant stress. *Am. J. Bot.* 80 239–243. 10.1002/j.1537-2197.1993.tb13796.x

[B17] Carter G. A. Knapp A. K. (2001). Leaf optical properties in higher plants: linking spectral characteristics to stress and chlorophyll concentration. *Am. J. Bot.* 88 677–684. 10.2307/2657068 11302854

[B18] Chaffei C. Pageau K. Suzuki A. Gouia H. Ghorbel M. H. Masclaux-Daubresse C. (2004). Cadmium toxicity induced changes in nitrogen management in *Lycopersicon esculentum* leading to a metabolic safeguard through an amino acid storage strategy. *Plant Cell Physiol.* 45 1681–1693. 10.1093/pcp/pch192 15574844

[B19] Clemente S. H. Rodrigues L. R. Ponce R. Varela S. A. Magalhães S. (2016). Incomplete species recognition entails few costs in spider mites, despite first-male precedence. *Behav. Ecol. Sociobiol.* 70 1161–1170. 10.1007/s00265-016-2124-0

[B20] Clemente S. H. Santos I. Ponce R. Rodrigues L. R. Varela S. A. Magalhães S. (2018). Despite reproductive interference, the net outcome of reproductive interactions among spider mite species is not necessarily costly. *Behav. Ecol.* 29 321–327. 10.1093/beheco/arx161

[B21] Couée I. Sulmon C. Gouesbet G. El Amrani A. (2006). Involvement of soluble sugars in reactive oxygen species balance and responses to oxidative stress in plants. *J. Exp. Bot.* 57 449–459. 10.1093/jxb/erj027 16397003

[B22] Das P. Samantaray S. Rout G. R. (1997). Studies on cadmium toxicity in plants: a review. *Environ. Pollut.* 98 29–36. 10.1016/S0269-7491(97)00110-3 15093342

[B23] Davis M. A. Pritchard S. G. Boyd R. S. Prior S. A. (2001). Developmental and induced responses of nickel-based and organic defences of the nickel-hyperaccumulating shrub, *Psychotria douarrei*. *New Phytol.* 150 49–58. 10.1046/j.1469-8137.2001.00067.x

[B24] de Carvalho R. C. da Silva A. B. Branquinho C. da Silva J. M. (2015). Influence of dehydration rate on cell sucrose and water relations parameters in an inducible desiccation tolerant aquatic bryophyte. *Environ. Exp. Bot.* 120 18–22. 10.1016/j.envexpbot.2015.07.002

[B25] de Souza M. P. Huang C. P. A. Chee N. Terry N. (1999). Rhizosphere bacteria enhance the accumulation of selenium and mercury in wetland plants. *Planta* 209 259–263. 10.1007/s004250050630 10436229

[B26] Devi R. Munjral N. Gupta A. K. Kaur N. (2007). Cadmium induced changes in carbohydrate status and enzymes of carbohydrate metabolism, glycolysis and pentose phosphate pathway in pea. *Environ. Exp. Bot.* 61 167–174. 10.1016/j.envexpbot.2007.05.006

[B27] Dong J. Wu F. Zhang G. (2006). Influence of cadmium on antioxidant capacity and four microelement concentrations in tomato seedlings (*Lycopersicon esculentum*). *Chemosphere* 64 1659–1666. 10.1016/j.chemosphere.2006.01.030 16497361

[B28] Farinati S. DalCorso G. Bona E. Corbella M. Lampis S. Cecconi D. (2009). Proteomic analysis of *Arabidopsis halleri* shoots in response to the heavy metals cadmium and zinc and rhizosphere microorganisms. *Proteomics* 9 4837–4850. 10.1002/pmic.200900036 19810031

[B29] Farinati S. DalCorso G. Panigati M. Furini A. (2011). Interaction between selected bacterial strains and *Arabidopsis halleri* modulates shoot proteome and cadmium and zinc accumulation. *J. Exp. Bot.* 62 3433–3447. 10.1093/jxb/err015 21357773 PMC3130167

[B30] Fones H. N. Eyles C. J. Bennett M. H. Smith J. A. C. Preston G. M. (2013). Uncoupling of reactive oxygen species accumulation and defence signalling in the metal hyperaccumulator plant *Noccaea caerulescens*. *New Phytol.* 199 916–924. 10.1111/nph.12354 23758201

[B31] Freeman J. L. Lindblom S. D. Quinn C. F. Fakra S. Marcus M. A. Pilon-Smits E. A. (2007). Selenium accumulation protects plants from herbivory by Orthoptera via toxicity and deterrence. *New Phytol.* 175 490–500. 10.1111/j.1469-8137.2007.02119.x 17635224

[B32] Godinho D. P. Janssen A. Dias T. Cruz C. Magalhães S. (2016). Down-regulation of plant defence in a resident spider mite species and its effect upon con-and heterospecifics. *Oecologia* 180 161–167. 10.1007/s00442-015-3434-z 26369779

[B33] Gomes M. Marques T. Soares A. (2013). Cadmium effects on mineral nutrition of the Cd-hyperaccumulator *Pfaffia glomerata*. *Biologia* 68 223–230. 10.2478/s11756-013-0005-9

[B34] Gratão P. L. Monteiro C. C. Antunes A. M. Peres L. E. P. Azevedo R. A. (2008). Acquired tolerance of tomato (*Lycopersicon esculentum* cv. Micro-Tom) plants to cadmium-induced stress. *Ann. Appl. Biol.* 153 321–333. 10.1111/j.1744-7348.2008.00299.x

[B35] Hanson B. Garifullina G. F. Lindblom S. D. Wangeline A. Ackley A. Kramer K. (2003). Selenium accumulation protects *Brassica juncea* from invertebrate herbivory and fungal infection. *New Phytol.* 159 461–469. 10.1046/j.1469-8137.2003.00786.x 33873368

[B36] Hartke S. Da Silva A. A. de Moraes M. G. (2013). Cadmium accumulation in tomato cultivars and its effect on expression of metal transport-related genes. *Bull. Environ. Contam. Toxicol.* 90 227–232. 10.1007/s00128-012-0899-x 23224767

[B37] Herms D. A. Mattson W. J. (1992). The dilemma of plants: to grow or defend. *Q. Rev. Biol.* 67 283–335. 10.1086/417659

[B38] Hörger A. C. Fones H. N. Preston G. (2013). The current status of the elemental defense hypothesis in relation to pathogens. *Front. Plant Sci.* 4:395. 10.3389/fpls.2013.00395 24137169 PMC3797420

[B39] Izaguirre M. M. Mazza C. A. SvatoŠ A. Baldwin I. T. BallarÉ C. L. (2007). Solar ultraviolet-B radiation and insect herbivory trigger partially overlapping phenolic responses in *Nicotiana attenuata* and *Nicotiana longiflora*. *Ann. Bot.* 99 103–109. 10.1093/aob/mcl226 17210605 PMC2802969

[B40] Jhee E. M. Boyd R. S. Eubanks M. D. (2005). Nickel hyperaccumulation as an elemental defense of *Streptanthus polygaloides* (Brassicaceae): influence of herbivore feeding mode. *New Phytol.* 168 331–344. 10.1111/j.1469-8137.2005.01504.x 16219073

[B41] Joutei A. B. Roy J. Van Impe G. Lebrun P. (2000). Effect of elevated CO2 on the demography of a leaf-sucking mite feeding on bean. *Oecologia* 123 75–81. 10.1007/s004420050991 28308746

[B42] Kant M. R. Ament K. Sabelis M. W. Haring M. A. Schuurink R. C. (2004). Differential timing of spider mite-induced direct and indirect defenses in tomato plants. *Plant Physiol.* 135 483–495. 10.1104/pp.103.038315 15122016 PMC429400

[B43] Karban R. Myers J. H. (1989). Induced plant responses to herbivory. *Annu. Rev. Ecol. Syst.* 20 331–348. 10.1146/annurev.es.20.110189.001555

[B44] Kastori R. Petrović M. Petrović N. (1992). Effect of excess lead, cadmium, copper, and zinc on water relations in sunflower. *J. Plant Nutr.* 15 2427–2439. 10.1080/01904169209364485

[B45] Kazemi-Dinan A. Sauer J. Stein R. J. Krämer U. Müller C. (2015). Is there a trade-off between glucosinolate-based organic and inorganic defences in a metal hyperaccumulator in the field? *Oecologia* 178 369–378. 10.1007/s00442-014-3218-x 25582869

[B46] Kazemi-Dinan A. Thomaschky S. Stein R. J. Kraemer U. Mueller C. (2014). Zinc and cadmium hyperaccumulation act as deterrents towards specialist herbivores and impede the performance of a generalist herbivore. *New Phytol.* 202 628–639. 10.1111/nph.12663 24383491

[B47] Khan S. Khan N. N. (1983). Influence of lead and cadmium on the growth and nutrient concentration of tomato (*Lycopersicum esculentum*) and egg-plant (*Solanum melongena*). *Plant Soil* 74 387–394. 10.1007/BF02181356

[B48] Konopka J. K. Hanyu K. Macfie S. M. McNeil J. N. (2013). Does the response of insect herbivores to cadmium depend on their feeding strategy? *J. Chem. Ecol.* 39 546–554. 10.1007/s10886-013-0273-4 23525953

[B49] Kozlov M. V. (2003). Density fluctuations of the leafminer *Phyllonorycter strigulatella* (Lepidoptera: Gracillariidae) in the impact zone of a power plant. *Environ. Pollut.* 121 1–10. 10.1016/S0269-7491(02)00213-0 12475055

[B50] Kuboi T. Noguchi A. Yazaki J. (1986). Family-dependent cadmium accumulation characteristics in higher plants. *Plant Soil* 92 405–415. 10.1007/BF02372488

[B51] Larbi A. Morales F. Abadía A. Gogorcena Y. Lucena J. J. Abadía J. (2002). Effects of Cd and Pb in sugar beet plants grown in nutrient solution: induced Fe deficiency and growth inhibition. *Funct. Plant Biol.* 29 1453–1464. 10.1071/FP02090 32688745

[B52] Li C. Williams M. M. Loh Y. T. Lee G. I. Howe G. A. (2002). Resistance of cultivated tomato to cell content-feeding herbivores is regulated by the octadecanoid-signaling pathway. *Plant Physiol.* 130 494–503. 10.1104/pp.005314 12226528 PMC166581

[B53] Lin Y. L. Chao Y. Y. Kao C. H. (2010). Exposure of rice seedlings to heat shock protects against subsequent Cd-induced decrease in glutamine synthetase activity and increase in specific protease activity in leaves. *J. Plant Physiol.* 167 1061–1065. 10.1016/j.jplph.2010.03.002 20399533

[B54] López-Millán A. F. Sagardoy R. Solanas M. Abadía A. Abadía J. (2009). Cadmium toxicity in tomato (*Lycopersicon esculentum*) plants grown in hydroponics. *Environ. Exp. Bot.* 65 376–385. 10.1016/j.envexpbot.2008.11.010

[B55] Martens S. N. Boyd R. S. (1994). The ecological significance of nickel hyperaccumulation: a plant chemical defense. *Oecologia* 98 379–384. 10.1007/BF00324227 28313915

[B56] Mishra B. Sangwan R. S. Mishra S. Jadaun J. S. Sabir F. Sangwan N. S. (2014). Effect of cadmium stress on inductive enzymatic and nonenzymatic responses of ROS and sugar metabolism in multiple shoot cultures of Ashwagandha (*Withania somnifera* Dunal). *Protoplasma* 251 1031–1045. 10.1007/s00709-014-0613-4 24510215

[B57] Musser R. O. Hum-Musser S. M. Eichenseer H. Peiffer M. Ervin G. Murphy J. B. (2002). Herbivory: caterpillar saliva beats plant defences. *Nature* 416 599–600. 10.1038/416599a 11948341

[B58] Oksanen J. Blanchet F. G. Kindt R. Legendre P. Minchin P. R. O’hara R. B. (2013). *Package ‘Vegan’. Community Ecology Package, Version, 2.0–5*. Avaliable at: https://CRAN.R-project.org/package=vegan.

[B59] Paulo J. T. Godinho D. P. Silva A. Branquinho C. Magalhães S. (2018). Suppression of plant defenses by herbivorous mites is not associated with adaptation to host plants. *Int. J. Mol. Sci.* 19:1783. 10.3390/ijms19061783 29914126 PMC6032058

[B60] Pena L. B. Pasquini L. A. Tomaro M. L. Gallego S. M. (2006). Proteolytic system in sunflower (*Helianthus annuus* L.) leaves under cadmium stress. *Plant Sci.* 171 531–537. 10.1016/j.plantsci.2006.06.003 25193651

[B61] Pereira A. S. Cortez P. A. de Almeida A. A. F. Prasad M. N. V. França M. G. C. da Cunha M. (2017). Morphology, ultrastructure, and element uptake in *Calophyllum brasiliense* Cambess.(*Calophyllaceae J*. Agardh) seedlings under cadmium exposure. *Environ. Sci. Poll. Res.* 24 15576–15588. 10.1007/s11356-017-9187-y 28516356

[B62] Petit C. M. Ringoet A. Myttenaere C. (1978). Stimulation of cadmium uptake in relation to the cadmium content of plants. *Plant Physiol.* 62 554–557. 10.1104/pp.62.4.554 16660557 PMC1092169

[B63] Petit C. M. Van de Geijn S. C. (1978). In vivo measurement of cadmium (115mCd) transport and accumulation in the stems of intact tomato plants (*Lycopersicon esculentum*, Mill.). *Planta* 138 137–143. 10.1007/BF00391170 24414008

[B64] Pollard A. J. (2000). Metal hyperaccumulation: a model system for coevolutionary studies. *New Phytol.* 146 179–181. 10.1046/j.1469-8137.2000.00651.x 33862980

[B65] Poschenrieder C. Cabot C. Martos S. Gallego B. Barceló J. (2013). Do toxic ions induce hormesis in plants? *Plant Sci.* 212 15–25. 10.1016/j.plantsci.2013.07.012 24094050

[B66] Poschenrieder C. Tolra R. Barcelo J. (2006). Can metals defend plants against biotic stress? *Trends Plant Sci.* 11 288–295. 10.1016/j.tplants.2006.04.007 16697693

[B67] Quinn C. F. Freeman J. L. Reynolds R. J. Cappa J. J. Fakra S. C. Marcus M. A. (2010). Selenium hyperaccumulation offers protection from cell disruptor herbivores. *BMC Ecol.* 10:19. 10.1186/1472-6785-10-19 20799959 PMC2940900

[B68] Roberts M. R. Paul N. D. (2006). Seduced by the dark side: integrating molecular and ecological perspectives on the influence of light on plant defence against pests and pathogens. *New Phytol.* 170 677–699. 10.1111/j.1469-8137.2006.01707.x 16684231

[B69] Rodrigues C. I. Maia R. Miranda M. Ribeirinho M. Nogueira J. M. F. Máguas C. (2009). Stable isotope analysis for green coffee bean: a possible method for geographic origin discrimination. *J. Food Compost. Anal.* 22 463–471. 10.1016/j.jfca.2008.06.010

[B70] Rosa M. Prado C. Podazza G. Interdonato R. González J. A. Hilal M. (2009). Soluble sugars: metabolism, sensing and abiotic stress: a complex network in the life of plants. *Plant Signal. Behav.* 4 388–393. 10.4161/psb.4.5.8294 19816104 PMC2676748

[B71] Santos A. A. D. Deoti J. R. Müller G. Dário M. G. Stambuk B. U. Alves Junior S. L. (2017). Microwell plate-based method for the determination of reducing sugars with the DNS reagent. *Brazil. J. Food Technol.* 20 1–9. 10.1590/1981-6723.11315

[B72] Sarmento R. A. Lemos F. Bleeker P. M. Schuurink R. C. Pallini A. Oliveira M. G. A. (2011). A herbivore that manipulates plant defence. *Ecol. Lett.* 14 229–236. 10.1111/j.1461-0248.2010.01575.x 21299823 PMC3084520

[B73] Scheirs J. Vandevyvere I. Wollaert K. Blust R. De Bruyn L. (2006). Plant-mediated effects of heavy metal pollution on host choice of a grass miner. *Environ. Pollut.* 143 138–145. 10.1016/j.envpol.2005.11.001 16360250

[B74] Shackira A. M. Puthur J. T. (2017). Enhanced phytostabilization of cadmium by a halophyte—*Acanthus ilicifolius* L. *Int. J. Phytoremediation* 19 319–326. 10.1080/15226514.2016.1225284 27593613

[B75] Siddhu G. Sirohi D. S. Kashyap K. Khan I. A. Khan M. A. (2008). Toxicity of cadmium on the growth and yield of *Solanum melongena* L. *J. Environ. Biol.* 29 853–857. 19297979

[B76] Sridhar B. M. Han F. X. Diehl S. V. Monts D. L. Su Y. (2007). Spectral reflectance and leaf internal structure changes of barley plants due to phytoextraction of zinc and cadmium. *Int. J. Remote Sens.* 28 1041–1054. 10.1080/01431160500075832

[B77] Stolpe C. Krämer U. Müller C. (2017). Heavy metal (hyper) accumulation in leaves of *Arabidopsis halleri* is accompanied by a reduced performance of herbivores and shifts in leaf glucosinolate and element concentrations. *Environ. Exp. Bot.* 133 78–86. 10.1016/j.envexpbot.2016.10.003

[B78] Sun X. Zhang J. Zhang H. Zhang Q. Ni Y. Chen J. (2009). Glucosinolate profiles of *Arabidopsis thaliana* in response to cadmium exposure. *Water Air Soil Pollut.* 200 109–117. 10.1007/s11270-008-9897-3

[B79] Tewes L. J. Stolpe C. Kerim A. Krämer U. Müller C. (2018). Metal hyperaccumulation in the Brassicaceae species *Arabidopsis halleri* reduces camalexin induction after fungal pathogen attack. *Environ. Exp. Bot.* 153 120–126. 10.1016/j.envexpbot.2018.05.015

[B80] Tolra R. P. Poschenrieder C. Alonso R. Barceló D. Barceló J. (2001). Influence of zinc hyperaccumulation on glucosinolates in *Thlaspi caerulescens*. *New Phytol.* 151 621–626. 10.1046/j.0028-646x.2001.00221.x 33853264

[B81] Vesk P. A. Reichman S. M. (2009). Hyperaccumulators and herbivores—a Bayesian meta-analysis of feeding choice trials. *J. Chem. Ecol.* 35 289–296. 10.1007/s10886-009-9607-7 19242760

[B82] Wahid A. Ghani A. Ali I. Ashraf M. Y. (2007). Effects of cadmium on carbon and nitrogen assimilation in shoots of mungbean [*Vigna radiata* (L.) Wilczek] seedlings. *J. Agron. Crop Sci.* 193 357–365. 10.1111/j.1439-037X.2007.00270.x

[B83] Walling L. L. (2000). The myriad plant responses to herbivores. *J. Plant Growth Regul.* 19 195–216. 10.1007/s003440000026 11038228

[B84] Wang W. Vinocur B. Altman A. (2003). Plant responses to drought, salinity and extreme temperatures: towards genetic engineering for stress tolerance. *Planta* 218 1–14. 10.1007/s00425-003-1105-5 14513379

[B85] Wermelinger B. Oertli J. J. Delucchi V. (1985). Effect of host plant nitrogen fertilization on the biology of the two-spotted spider mite, *Tetranychus urticae*. *Entomol. Exp. Appl.* 38 23–28. 10.1111/j.1570-7458.1985.tb03493.x

[B86] White T. T. (1984). The abundance of invertebrate herbivores in relation to the availability of nitrogen in stressed food plants. *Oecologia* 63 90–105. 10.1007/BF00379790 28311171

[B87] Ximénez-Embún M. G. Castañera P. Ortego F. (2017). Drought stress in tomato increases the performance of adapted and non-adapted strains of Tetranychus urticae. *J. Insect Physiol.* 96 73–81. 10.1016/j.jinsphys.2016.10.015 27789296

[B88] Ximénez-Embún M. G. Ortego F. Castañera P. (2016). Drought-stressed tomato plants trigger bottom–up effects on the invasive *Tetranychus evansi*. *PLoS One* 11:e0145275. 10.1371/journal.pone.0145275 26735490 PMC4703393

[B89] Zélé F. Santos I. Olivieri I. Weill M. Duron O. Magalhães S. (2018). Endosymbiont diversity and prevalence in herbivorous spider mite populations in South-Western Europe. *FEMS Microbiol. Ecol.* 94:fiy015. 10.1093/femsec/fiy015 29390142

[B90] Zvereva E. L. Kozlov M. V. Neuvonen S. (1995). Population density and performance of Melasoma lapponica (Coleoptera: Chrysomelidae) in surroundings of smelter complex. *Environ. Entomol.* 24 707–715. 10.1093/ee/24.3.707

